# A quantitative analysis of sources of changes in government expenditures on health, 2000 to 2015: what can we learn from experience to date?

**DOI:** 10.12688/gatesopenres.12900.2

**Published:** 2021-08-26

**Authors:** Helen Saxenian, Ipchita Bharali, Osondu Ogbuoji, Gavin Yamey

**Affiliations:** 1 The Center for Policy Impact in Global Health, Duke Global Health Institute, Durham, NC, 27708, USA

**Keywords:** health, health financing, domestic resource mobilization, universal health coverage

## Abstract

**Background: **Achieving universal health coverage (UHC) requires increased domestic financing of health by low-income countries (LICs) and middle-income countries (MICs). It is critical to understand how much governments have devoted to health from domestic sources and how much growth might be realistic over time.

**Methods:** Using data from WHO’s Global Health Expenditure Database, we examined how the composition of current health expenditure changed by financing source and the sources of growth in health expenditures from 2000-2015 across different income groups. We disaggregated how much growth in government expenditures on health from domestic sources was due to economic growth, growth in government spending as a share of GDP, and reallocations in government expenditures towards health.

**Results:** Lower MICs (LMICs) and upper MICs (UMICs), as a group, saw a significant reduction in out-of-pocket expenditures and a significant growth in government expenditures on health from domestic sources as a share of current health expenditures over the period. This trend indicates likely progress in the pathway to UHC. For LICs, these trends were more muted. Growth in government expenditure on health from domestic sources was driven primarily by economic growth in LICs, LMICs, and UMICs. Growth in government expenditure on health due to increased government spending as a share of GDP was high in UMICs. For the high-income country group, where economic growth was relatively slower and government spending was already high with strong tax bases, the largest driver of growth in government expenditure on health from domestic sources was reallocation of the government budget towards health.

**Conclusions:** Dialogue on domestic resource mobilization needs to emphasize overall economic growth and growth in the government spending as a share of GDP as well as the share of health in the government budget.

## Background

Many low-income countries (LICs) and middle-income countries (MICs) have committed to the ambitious health-related Sustainable Development Goals (SDGs), such as the achievement of universal health coverage (UHC), which will require additional resources for health, alongside other measures. For example, Stenberg and colleagues estimate that it will cost an additional $371 billion annually across 67 LICs and MICs (those representing 95% of the population of all LICs and MICs) to strengthen health systems to achieve the health-related SDGs ^1^. At the same time, some MICs are now transitioning out of grant or concessional external finance mechanisms, such as support from Gavi, the Vaccine Alliance, The Global Fund to Fight AIDS, Tuberculosis and Malaria, and World Bank International Development Association credits. Such MICs need to replace these sources of external finance with domestic financing. Given these twin shifts in global health—the adoption of highly ambitious health goals that necessitate scaled up financing, coupled with the transition of MICs away from development assistance for health (DAH)—there is intense interest in understanding the role of domestic finance for health in LICs and MICs in the SDGs era.

More specifically, it has become critical to understand how much governments have devoted to health from their own sources and how much growth might be realistic over time. This study aimed to elucidate these trends by examining the sources of financing for health from 2000 to 2015 in LICs and MICs, with a focus on government expenditure from domestic sources. Our goal was to understand the recent experience of LICs and MICs in financing their health sectors in order to better estimate the possible scope for future domestic resource mobilization.

This analysis was feasible because of the major effort that the World Health Organization (WHO) made to update and reorganize its Global Health Expenditure Database (GHED). The GHED introduced major improvements in the December 2017 data release to follow the System of Health Accounts 2011 ^2^ (SHA2011), which “tracks all health spending in a given country over a defined period of time regardless of the entity or institution that financed and managed that spending.” Health expenditure estimates for 2000 onwards were revised to disaggregate expenditures by financing *source* (public, private, and external) as well as by financing scheme (the arrangements through which spending is made). The reorganization also separated out *current* expenditure (such as wages, goods, and services) from *capital* expenditure (such as investment in more durable equipment and infrastructure).

Our study complements the analysis that the WHO published on the new GHED, an analysis called New Perspectives on Global Health Spending for Universal Health Coverage ^3^. Our study goes into more detail on financing sources, particularly the changes in government expenditure on health, relative to other indicators. We use data from the new GHED to explore (1) how government expenditure on health from domestic sources grew relative to both gross domestic product (GDP) and general government expenditure (GGE), and (2) how these indicators changed across income groups. Our findings can be used to assess country progress against existing targets for domestic health spending, and to develop scenarios of domestic resource projections going forward. Our analysis also complements a study that the World Bank published in December 2018 entitled the Intertemporal Dynamics of Public Financing for Universal Health Coverage: Accounting for Fiscal Space Across Countries ^4^. The World Bank study also explored how financing sources grew.

In addition to mobilizing new resources for health, another way to expand fiscal space (budgetary room) for health is through efficiency gains. Our study does *not* examine such gains.

## Methods

Our Methods sections have seven key steps. We first describe the GHED, including the sources of health expenditure reported in this database, since this is the key source of data for our study. Second, we explain the rationale for our sample size. Third, we describe how we grouped countries by income level. Fourth, we describe the completeness of the data used in our study. Fifth, we lay out the scope of our study and delineate the specific research questions. Sixth, we summarize how we calculated the key metrics in our study. Finally, we explain how we conducted a decomposition analysis using the data available within the GHED to identify the key drivers of growth in general government health expenditures.

### The Global Health Expenditure Database

The GHED was updated in December 2017 to disaggregate expenditures by financing source. The update furthermore disaggregated expenditures by financing arrangement and separated out recurrent (or current) expenditure from capital expenditure. Unless clearly stated as capital expenditures, the health expenditure indicators include only current expenditure. Current expenditures are made up of expenditures on resources that are consumed within one year, including wages, goods, and services. Capital expenditures are expenditures on assets such as buildings and equipment that have a working life of one year or longer. This distinction is intended to make current expenditure estimates more comparable year on year, as capital expenditures tend to be “lumpy.” If capital expenditures and current expenditures were combined, an increase or fall in health expenditure could be due in part to the timing of infrastructure projects.

**Figure 1.**
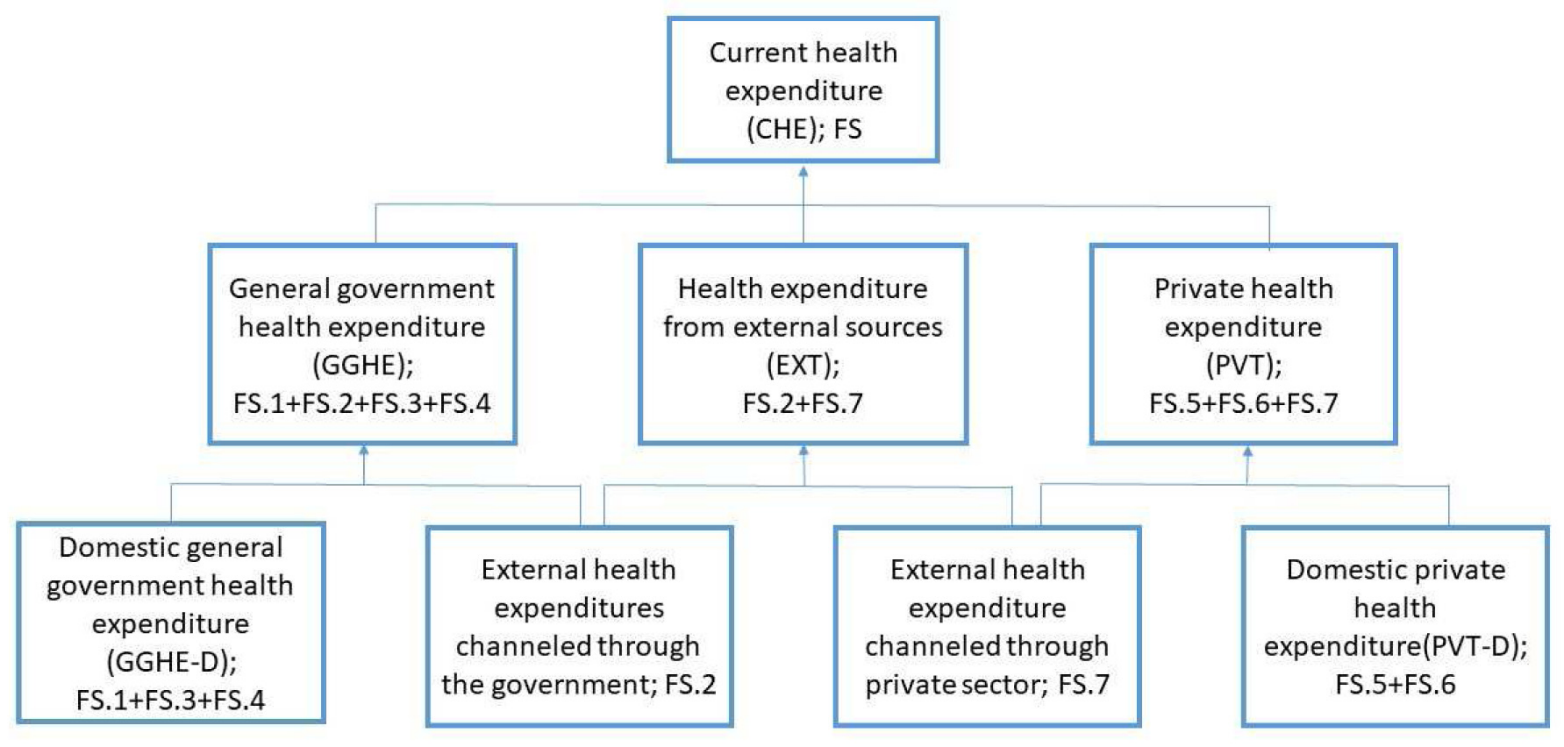
Sources of health expenditures reported in the Global Health Expenditure Database.

The indicator current health expenditure consists of domestic general government expenditure (GGHE-D), health expenditure from external sources (EXT), and domestic private health expenditure (PVT-D) ( Figure 1). The paper follows the WHO definitions of sources of financing. Domestic general government health expenditure refers to expenditures in the form of all internals transfers and grants, subsidies to voluntary health insurance beneficiaries, and non-government financing agencies, as well as compulsory prepayment and social health insurance contributions. One point that is unclear in the GHED is how well external assistance is disaggregated between capital and current expenditure.

Figure 1 provides a breakdown of the sources of expenditure data, with the terminology used in the GHED. Further information is provided in WHO’s Technical brief ^5^ on the Indicators published on the World Health Organization’s Global Health Expenditure Database.

### Rationale for sample size

The GHED database contains 192 countries. We used two criteria to decide which countries would be included in our analysis:

- i. The population of the country in year 2015 was more than 600,000. This threshold was chosen to harmonize our analysis with the analysis reported in WHO’s global report *New Perspectives on Global Health Spending for Universal Health Coverage*; and
- ii. Data for the indicators GDP, GGHE-D, CHE, and out-of-pocket expenditures (OOP) were available for years 2000 and 2015.

Once countries with population less than 600,000 in year 2015 were eliminated, the sample size for our study was reduced from 192 to 159. After eliminating countries with missing values, the sample size further dropped from 159 to 125. Table 1 lists the countries included in the analysis by income group, and also lists the countries excluded and the corresponding rationale.

**Table 1.** List of countries included in analysis and list of countries excluded (and why).

Country income group (As per World Bank income classification in year 2000)	Countries
Low income	Angola, Armenia, Azerbaijan, Bangladesh, Benin, Burkina Faso, Burundi, Cambodia, Cameroon, Central African Republic, Chad, Congo, Côte d’Ivoire, DRC, Eritrea, Ethiopia, Georgia, Ghana, Guinea, Guinea-Bissau, Haiti, India, Indonesia, Kenya, Kyrgyzstan, Lao People’s Democratic Republic, Lesotho, Liberia, Madagascar, Malawi, Mali, Mauritania, Republic of Moldova, Mongolia, Mozambique, Myanmar, Nepal, Nicaragua, Niger, Nigeria, Pakistan, Rwanda, Senegal, Sierra Leone, Sudan, Tajikistan, United Republic of Tanzania, Togo, Uganda, Ukraine, Uzbekistan, Viet Nam, Yemen, Zambia
Lower middle income	Albania, Algeria, Belarus, Bolivia Plurinational States of, Bosnia and Herzegovina, Bulgaria, China, Colombia, Djibouti, Dominican Republic, Ecuador, Egypt, El Salvador, Equatorial Guinea, Fiji, Guatemala, Guyana, Honduras, Iran, Jamaica, Jordan, Kazakhstan, Latvia, Lithuania, The former Yugoslav Republic of Macedonia, Morocco, Namibia, Papua New Guinea, Paraguay, Peru, Philippines, Romania, Russian Federation, Sri Lanka, Swaziland, Thailand, Tunisia, Turkmenistan
Upper middle income	Argentina, Bahrain, Botswana, Brazil, Chile, Costa Rica, Croatia, Gabon, Republic of Korea, Lebanon, Malaysia, Mauritius, Oman, Panama, Saudi Arabia, South Africa, Trinidad and Tobago, Uruguay, Venezuela (Bolivarian Republic of)
High income	Canada, Cyprus, Denmark, Finland, Germany, Italy, Kuwait, Portugal, Singapore, United Arab Emirates, United Kingdom, United States of America
Unclassified (Newly independent states formed after 2000)	Montenegro, Serbia
Countries excluded by reason of exclusion	Countries
Population less than 600,000 as of 2015	Andorra, Antigua and Barbuda, Bahamas, Barbados, Belize, Brunei Darussalam, Cabo Verde Republic of, Cook Islands, Dominica, Gambia, Grenada, Kiribati, Luxembourg, Maldives, Malta, Marshall Islands, Micronesia (Federated States of), Monaco, Nauru, Niue, Palau, Saint Kitts and Nevis, Saint Lucia, Saint Vincent and the Grenadines, Samoa, San Marino, Sao Tome and Principe, Seychelles, Solomon Islands, Suriname, Tonga, Tuvalu, Vanuatu
Data on either GDP, GGE, GGHE-D, CHE, or OOP is missing	Afghanistan, Australia, Austria, Belgium, Bhutan, Comoros, Estonia, France, Greece, Hungary, Iceland, Japan, Iraq, Ireland, Israel, Libya, Mexico, Netherlands, New Zealand, Norway, Poland, Qatar, Slovakia, Slovenia, Somalia, South Sudan, Spain, Sweden, Switzerland, Syria, Timor-Leste, Turkey, Zimbabwe

### Grouping of countries into income classifications

For grouping countries in the analysis, we used the World Bank analytical income classification system, which groups countries into low-income countries (LICs), lower-middle-income countries (LMICs), upper-middle-income countries (UMICs), and high-income countries (HICs). For our analysis, we classified each country as a LIC, LMIC, UMIC, or HIC at the *start* of the period of interest to us, in this case the year 2000, and held the groups constant from 2000 to 2015. In contrast, both the WHO ^3^ and World Bank ^4^ studies classified countries by their income status at the *end* of the period, or by 2015. Given the lags in data availability, the World Bank uses calendar year 2000 income data for its analytical income classification in its fiscal year (FY) 2002 (FY02). For this paper, we use the classification for FY02, based on 2000 income data.

As context, over the 15-year period, there was significant movement of countries across income categories. Every year, the World Bank updates the income thresholds in order to keep them constant in real terms and reclassifies countries based on their most recent Gross National Income (GNI) per capita. The total number of economies classified grew from 205 to 218 over the study period 2000–2015 due to the inclusion of newly independent states (Kosovo, Montenegro, Serbia, South Sudan, and Timor-Leste) as well as several small economies that had not previously been classified. Due to income growth, many countries changed income classification from 2000 to 2015. A total of 33 countries moved from LIC to LMIC or UMIC, and one (new) country, South Sudan was not classified until year 2011. The largest country that moved out of the LIC category was India. Another 31 countries moved from LMIC to UMIC, the largest of which was China, while 18 countries moved from UMIC to HIC. The changes in terms of income classification of the world’s population are more dramatic than the changes in terms of number of countries. About 41% of the world’s population was living in LICs in 2000. This proportion fell to 8% by 2015 ( Appendix 1 ^6^). In 2000, 11% of the world’s population lived in UMICs, which rose to 35% by 2015.

According to their 2015 GNI p.c., only 31 countries were still in the LIC category by 2015, compared to 63 at the start of the study period in 2000 ( Appendix 2 ^6^). The rationale for defining income groups at the start of the period is because the purpose of the analysis is to understand how health financing evolved for the group of countries classified similarly at the start of the period, to avoid selection bias.

We have created two additional income groups, namely, LICs without India (as India was classified as an LIC at the start of the period) and LMICs without China (as China was classified as an LMIC at the start of the period), to study the changes in health expenditures across the respective income groups excluding these two large economies and also to study changes in these two economies individually.

### Data completeness and accuracy

Overall, the data on financing sources indicators are most complete for LICs and MICs. For the indicator GGHE-D as a percent of CHE, about 90% of LICs have data for all the years 2000 to 2015 and about 92% of MICs have a complete set of data. In contrast, only about 65% of HICs have complete data for the period 2000 to 2015.

The accuracy of data reported in the GHED varies on a country-to-country basis. National Health Account exercises form the core of a solid understanding of sources of financing at the country level. Some countries have completed national health accounts (NHA) exercises multiple times, and others have yet to conduct them. Most countries publish expenditure data, but others only provide budgeted data. For cases where NHA data is not regularly available, WHO has filled missing data gaps by using government budget data, interpolations and data from international sources such as the Organization for Economic Cooperation and Development’s Development Assistance Committee database, or through in-country consultations. Inevitably, some country data are much stronger than other country data.

### Scope and key research questions

This study first provides a general overview on changes in sources of financing, and then it more deeply analyzes the changes in GGHE-D. The GHED contains several other important macro indicators, including GGE and GDP. For the purposes of the analysis, we primarily compare the growth in GGHE-D to the growth in GDP, the growth in GGHE-D to the growth in GGE, and the change in the share of GGHE-D to GGE. The GGE is intended to capture all government (federal, state, local) current expenditure. Our study aims to answer three main questions based on the analysis of these key GHED indicators:

1) How did the composition of current health expenditure change during the period 2000 to 2015?
2) How did health expenditures from various sources change over this time period?
3) What were the main sources of growth in GGHE-D during this period?

### Calculation of metrics

To answer these three key questions, we looked at aggregate health expenditure estimates and ratios for the income groups defined above in sub-section (iii) and in two large economies, China and India. In this study, estimates are calculated for each income group as a unit. Our approach differs from the methodology used by WHO in their study *New Perspectives on Global Health Spending for Universal Health Coverage*; in that study, the WHO reports country-weighted averages. With country-weighted averages, a country such as India (1.3 billion population in 2015) is given the same weight in the LIC group as Comoros (800,000 population in 2015). In contrast, our method uses sums or aggregates across the countries in the income group, in order to generalize to the population in that income group. (For ratios, the aggregate ratios are calculated from the aggregates of the numerators and denominators). This approach is similar to how the World Bank and the IMF present aggregate economic indicators in their databases for different income or regions. In contrast, the World Bank study ^4^ *Intertemporal Dynamics of Public Financing for Universal Health Coverage: Accounting for Fiscal Space Across Countries,* like the WHO study ^3^, reported on income groups using country-weighted averages.

In order to aid comparison of our results with the WHO study and highlight the differences in methodologies of the two studies, some figures and tables in our study are intentionally designed to be similar to the ones used in the WHO study ( Figure 3 – Figure 5 and Appendix 3 ^6^).

**Figure 2.**
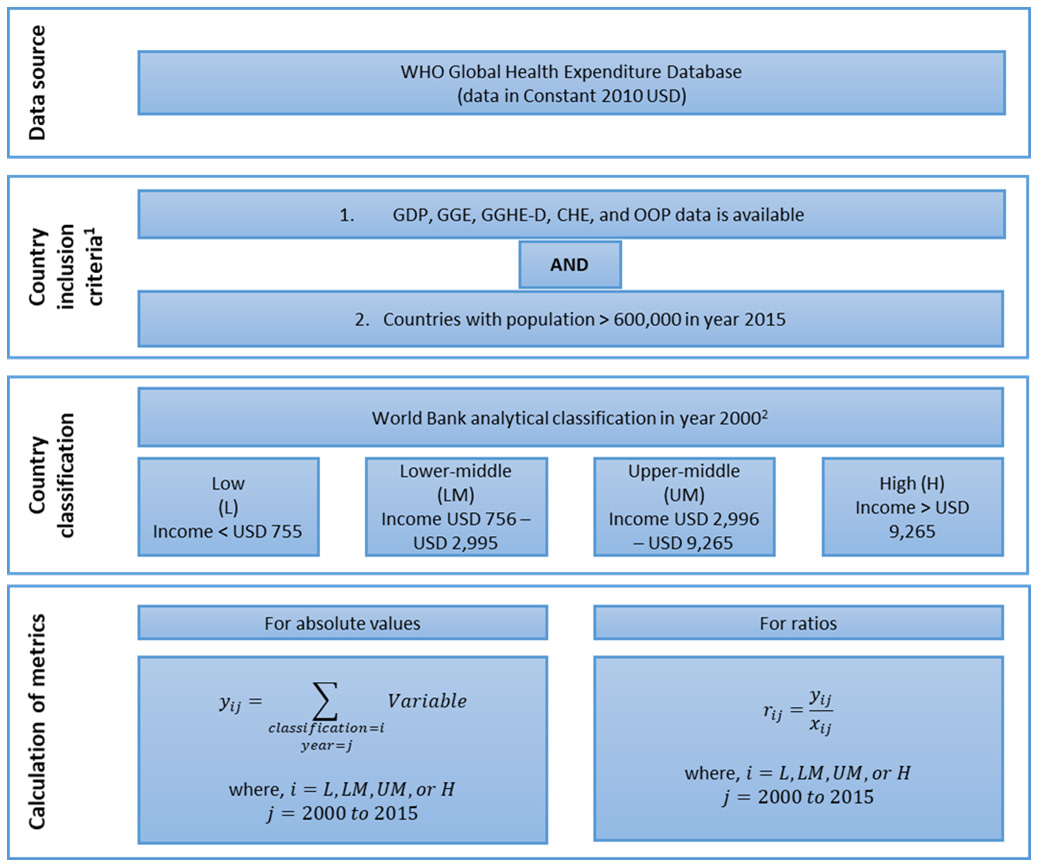
Flow chart showing data sources, country inclusion criteria, country classification by income, and calculation of key metrics. GDP, gross domestic product; GGE, general government expenditure; GGHE-D, domestic general government expenditure on health; CHE, current health expenditure; OOP, out-of-pocket expenditure.

Figure 2 is a flowchart that summarizes data sources, country inclusion criteria, country classification by income, and calculation of key metrics used in this study. It includes the formulae used for calculating absolute values and ratios. For a given income group *i*, the estimated health expenditure value for year *j* is the summation of reported values for all countries in that income category included in our analysis. Ratios are then calculated by simple division of the particular estimated values. The formula used for calculating annualized rate of growth is available under Table 3 and Table 4. The database and calculations used in this study can be viewed on OSF ^6^.

**Figure 3.**
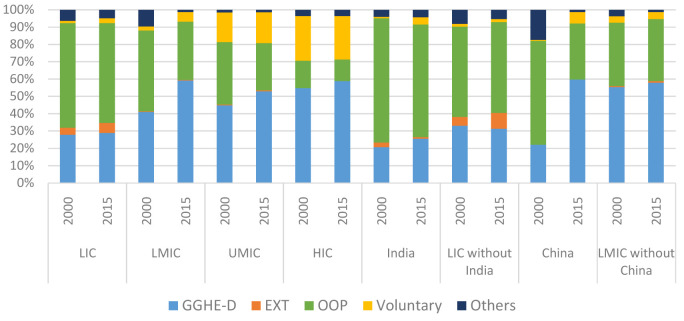
Composition of current health expenditure ^7^ by income group (% of total CHE), 2000 and 2015. GGHE-D, domestic general government expenditure on health; EXT, health expenditure from external sources; OOP, out-of-pocket expenditure.

**Figure 4.**
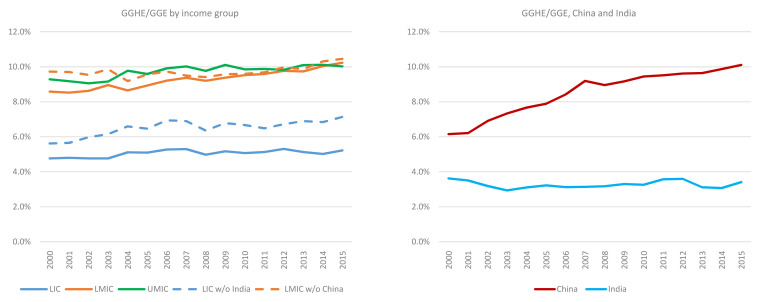
General government expenditure on health (GGHE)/general government expenditure (GGE) by income group, and separately for China and India, 2000 to 2015.

**Figure 5.**
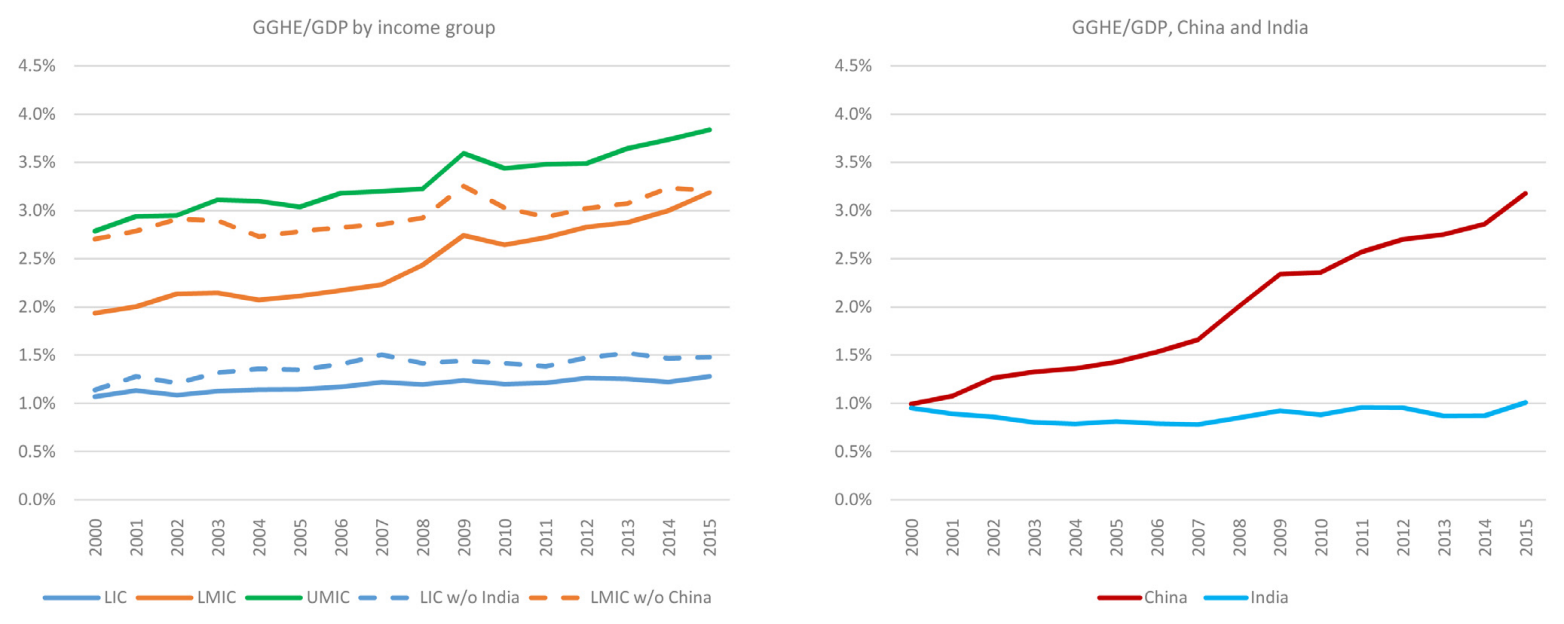
General government expenditure on health (GGHE)/ gross domestic product (GDP) by income group, and separately for China and India, 2000 to 2015.

### Decomposition of change in GGHE-D between 2000 and 2015

In addition to comparing trends in health expenditure estimates over the 2000 and 2015 time period, we have also conducted a decomposition analysis using the data available within the GHED to identify the key drivers of growth in general government health expenditures. This analysis is reported by income groups and separately for India and China, as they have large populations. We focus on three key drivers that determine the change in general government health expenditures from domestic sources:

### Figure 6. General government expenditure on health (GGHE)/gross domestic product (GDP) by income group, 2000 to 2015

i. GDP growth during the period
ii. Changes in government spending as a share of GDP (GGE/GDP)
iii. Changes in prioritization of health within government budgets measured by the ratio GGHE-D/GGE

This decomposition analysis is based on existing evidence that shows that the macroeconomic environment, level of government spending, and prioritization of the budget for health are key drivers of fiscal space for health ^7, 8^.

Mathematically, the level of domestic government health expenditures (GGHE-D) in year 2000 can be expressed as:

GGHE-D _2000_ = GDP _2000_ * (GGE/GDP) _2000_ * (GGHE-D/GGE) _2000_ ------------------------- (i)

If g is the rate of growth in GGHE-D between years 2000 and 2015,

GGHE-D _2015_ = (1+g)*GGHE-D _2000_ -----------------------------------(ii)

where g = (GGHE-D _2015_ - GGHE-D _2000_)/ GGHE-D _2000_ -------------------(iii)

From equations (i) and (ii), GGHE-D _2015_ can be expressed as:

(1+g)*GGHE-D _2000_ = (1+y)*GDP _2000_ * (1+e)* GGE/GDP _2000_ * (1+h)*GGHE-D/GGE _2000_ ------ (iv)

Where y, e, and h are the changes in GDP _,_ (GGE/GDP) _,_ and (GGHE-D/GGE) respectively and follow the same mathematical relationship shown in equation (iii) for the respective variables.

Taking the logarithm on both sides of equations (i) and (iv), the rate of change in GGHE-D between 2015 and 2000 can be expressed as:

ln (1+g) = ln(1+ y) +ln(1+ e) + ln (1+h) -------------------------- (v)

The derivation of equation (v) is provided in Appendix 5 ^6^.

In this paper, we find how much of the change in GGHE-D can be attributed to economic growth, changes in share of government spending to GDP, and allocation of government budget to health, using the mathematical relationship in equation (v). We report the results by income group.

While the results of the decomposition analysis can vary from country to country, the reporting by income group helps us identify whether the income groups have been creating more fiscal space for health, or whether growing government health expenditures are mostly spurred by economic growth. We have not looked at development assistance in this decomposition analysis as the focus of this paper is on general government health expenditures.

## Results

In this section, we divide the results of our study into five sub-sections. First, we present an overview of how the composition of CHE changed between 2000 and 2015. Second, we show these changes over time on a per capita basis. Third, we present annualized rates of growth in a number of key indicators. Fourth, we compare our findings on health expenditures from 2000–2015 with international targets on health spending (such as the 2001 Abuja declaration ^9^, which committed African Union countries to the target of allocating at least 15% of their annual budget to improve the health sector). Finally, we present results on the key drivers of the increases in GGHE-D from 2000–2015. More specifically, we show how much of the growth in GGHE-D, in real terms, from 2000 to 2015 was due to economic growth alone, expansion in public spending measured by GGE/GDP, or reallocation of GGE towards health measured by GGHE-D/GGE.

### Overview of how composition of CHE changed, 2000 and 2015

In this sub-section, we present the results of our analysis on the change in the composition of CHE from 2000–2015 from the five main sources of health financing

- GGHE-D
- EXT
- out-of-pocket expenditures (OOP), a component of domestic private health expenditure
- voluntary prepayment, a component of domestic private health expenditure, and
- other health expenditures, which refer to expenses by households (other than OOP), corporations, and nonprofit institutions, which are also categorized as FS.6.2, FS.6.3 and FS.6.nec in the GHED.

Figure 3 shows the changes in composition of CHE between 2000 and 2015 for income groups while Appendix 3 ^6^ shows the trend over time during this period. We also show China and India separately given their very large population size.

GGHE-D, domestic general government expenditure on health; EXT, health expenditure from external sources; OOP, out-of-pocket expenditure.

During this period, GGHE-D as a share of current health expenditure rose for all groups, and OOP spending as a share of current health expenditure—a key indicator of financial protection—fell across all income groups as well as China and India if examined separately. However, the increase (for GGHE-D) and decrease (for OOP) was negligible in the LIC group with only an increase in GGHE-D/CHE from 28% to 29% and a fall in OOP/CHE from 60% to 58% over the period 2000–2015. The increase of GGHE-D as a share of CHE was most pronounced among the LMICs, from 41% to 59% along with a fall in the share of OOP expenses from 47% to 34% of CHE.

Examined individually, given their large size, China and India are in stark contrast to each other in terms of improvement in share of GGHE-D over this period. While both China and India started with GGHE-D/CHE proportions of around 20% in 2000, China’s GGHE-D share of CHE increased three times to 60% by year 2015. By contrast, India’s GGHE-D share increased to around 30% in 2011, but fell to 26% of CHE in 2015. Along with government expenditure, China also saw an increase in voluntary insurance payments that reduced the share of OOP expenses by half.

### Changes in current health expenditure per capita, 2000 and 2015

Another way to examine changes in sources of expenditure is by changes over time on a per capita basis. Table 2 shows total current health expenditure per capita and per capita values from different financing sources across the income groups for 2000 and 2015. Current health expenditure per capita is the sum of GGHE-D per capita, EXT per capita, and private expenditure from domestic sources (PVT-D) per capita. The table also shows OOP expenditure per capita, typically the largest component of private expenditure from domestic sources. These numbers are expressed in constant 2010 US$ so they can be compared in real terms.

**Table 2.** Changes in current health expenditures per capita, 2000 and 2015 (in constant 2010 US$). Current health expenditure is the sum of domestic general government health expenditure (GGHE-D), health expenditure from external resources (EXT) and domestic private health expenditure (PVT-D); OOP is a subset of PVT-D.

Country groups	CHE per capita			GGHE-D per capita			EXT per capita			PVT per capita					
										OOP per capita			Other PVT-D per capita		
	2000	2015	% Change	2000	2015	% Change	2000	2015	% Change	2000	2015	% Change	2000	2015	% Change
**LIC**	31.2	68.7	119.7%	8.7	19.7	126.5%	1.3	4.1	225.5%	18.9	39.5	109.4%	2.4	5.3	120.3%
**LMIC**	114.6	333.3	190.8%	47.0	196.1	317.1%	0.4	1.1	197.7%	53.6	113.3	111.3%	13.6	22.9	67.8%
**UMIC**	543.5	857.4	57.8%	172.7	486.6	181.8%	2.1	3.6	75.6%	139.4	224.5	61%	229.4	142.8	-37.7%
**HIC**	3,895.0	5,213.1	33.8%	1,906.5	3,120.8	63.7%	0.0	0.4	***	524.4	635.9	21.3%	1464.1	1456.0	-0.6%
**China**	78.5	342.7	336.6%	17.4	204.9	1079.7%	N/A	0.0	N/A	47.0	111.0	136.0%	14.1	26.8	90.2%
**India**	31.0	68.0	119.4%	6.4	17.4	171.5%	0.8	0.6	-27%	22.2	44.2	99.1%	1.5	5.8	276.0%

GDP, gross domestic product; GGE, general government expenditure; GGHE-D, domestic general government health expenditure; CHE, current health expenditure; OOP, out-of-pocket expenditure. ***In case of high income countries, the percent change in external financing and resulting EXT per capita value saw a very large jump from 2009 to 2015 which is likely due to reclassification of health expenditures by high income countries supporting refugee populations in their home countries. LIC, low-income country; LMIC, lower-middle-income country; UMIC, upper-middle-income country; HIC, high-income country.

**Table 3.** Annual rate of growth, key indicators, 2000 to 2015.

Country group	No. of countries included in the analysis	Population	GDP	GGE	CHE	OOP	GGHE-D
**LIC: all**	54	1.8%	6.3%	6.8%	7.3%	7.0%	7.5%
**India**	1	1.5%	7.4%	8.3%	7.0%	6.2%	8.4%
**Excluding India**	53	2.1%	5.5%	5.6%	7.6%	7.6%	7.0%
**LMIC: all**	38	0.8%	7.1%	9.5%	8.2%	5.9%	10.8%
**China**	1	0.6%	9.7%	14.7%	11.0%	6.5%	18.6%
**Excluding China**	37	1.1%	4.1%	5.0%	5.2%	5.1%	5.5%
**UMIC**	19	1.2%	3.3%	4.9%	4.3%	4.4%	8.4%
**HIC**	12	0.7%	1.5%	1.9%	2.6%	2.0%	4.0%

Note: $Annualized\;Rate\;of\;Growth\;=\left({\left(\frac{X_{endyear}}{X_{baseyear}}\right)}^{\frac{1}{\text{endyear}-\text{baseyear}}}-1\right)*100$ GDP, gross domestic product; GGE, general government expenditure; GGHE-D, domestic general government health expenditure; CHE, current health expenditure; OOP, out-of-pocket expenditure.

**Table 4.** Annual rate of growth, key ratios and per capita values, 2000 to 2015.

Country group	No. of Countries included in the analysis	GGHE- D/GDP	GGHE- D/GGE	GGHE- D/CHE	OOP/CHE	GDP per capita	GGE per capita	GGHE-D per capita	CHE per capita
**LIC: all**	54	1.1%	0.5%	0.2%	-0.3%	4.4%	4.9%	5.6%	5.4%
**India**	1	0.9%	0.2%	1.4%	-0.6%	5.9%	6.7%	6.9%	5.4%
**Excluding** **India**	53	1.4%	1.2%	-0.5%	0.1%	3.4%	3.5%	4.9%	5.4%
**LMIC**	38	3.4%	1.2%	2.4%	-2.1%	6.3%	8.7%	10.0%	7.4%
**China**	1	8.1%	3.4%	6.9%	-4.0%	9.1%	14.0%	17.9%	10.3%
**Excluding** **China**	37	1.1%	0.4%	0.4%	-0.1%	3.0%	3.9%	4.4%	4.1%
**UMIC**	19	2.2%	0.5%	3.9%	0.1%	2.1%	3.6%	7.2%	3.1%
**HIC**	12	2.3%	1.5%	1.4%	-0.7%	0.8%	1.3%	3.3%	2.0%

Note:
$$Annualized\;Rate\;of\;Growth=\left({\left(\frac{X_{endyear}}{X_{baseyear}}\right)}^{\frac{1}{\text{endyear}-\mathrm{baseyear}}}-1\right)*100$$
GDP, gross domestic product; GGE, general government expenditure; GGHE-D, domestic general government health expenditure; CHE, current health expenditure; OOP, out-of-pocket expenditure.

Growth in overall current health expenditure per capita was fastest in China, where it increased fourfold. Since China started the period in the LMIC category, its fast growth had a large impact on this group of countries as a whole; across this group, expenditure per capita increased almost threefold. Health expenditure across the LIC group as a whole doubled from US$ 31 to US$ 69 per capita during this period. Growth was slowest in UMICs and HICs, where there was only 34% growth in the overall current health expenditure on a per capita basis. EXT per capita in LICs was only US$ 1.3 in year 2000, and grew to US$ 4 per capita by year 2015. EXT per capita in UMICs was higher than in LICs in 2000.

### Annualized rates of growth of key indicators

Annualized growth rates are useful to readily see how rapidly different sources of finance are growing relative to each other as well as relative to growth in population, GDP, and overall government expenditure. Some differentials in annualized growth rates are large, others in Table 3 may seem small, but over a 15-year time period they can result in significant changes in relative shares.

Population growth during this period was highest in LICs, at 1.8% per year compared to the lowest growth rate of 0.7% in HICs. GDP growth was highest in the LMIC group, at 7.1%, and lowest in HICs, at 1.5%. However, China’s extraordinary annualized growth of 9.7% over the period impacts the average for LMICs as a whole, as China was a LMIC at the start of the period. With China included, LMICs grew at 7.1% per year as a group, but when China is excluded, the growth rate was 4.1% per year. India also raised the overall growth rate of LICs as a whole, as India was a LIC at the start of the period. India’s fast growth of 7.4% per year in GDP pulled up the average for LICs to 6.3% per year. When India is excluded from the group, the growth rate for LICs was 5.5% per year. Economic growth in and of itself raises tax revenue, permitting more government expenditure on health.

Typically, as shown in a 2015 IMF report ^10^, as economies develop, the tax base expands and tax administration improves, and tax revenue as a share of GDP increases over time. Some countries that are highly dependent on natural resources, with undiversified economies, may be an important exception to this trend. In addition, a 2011 report ^11^ from the IMF Fiscal Affairs Department found that many resource-rich countries struggle with greater volatility in tax receipts compared to other countries. In addition to tax revenue from economic growth, tax revenue from an expanded tax base can provide a source for increased domestic government health expenditures. The GHED does not have data on tax revenue, but it does have data on GGE. Tax revenue and GGE are distinct concepts as expenditure can exceed revenue if governments run deficits. In all regions, GGE growth is faster than growth in GDP both in absolute and per capita terms, constant US$. The differential is largest in China, with GGE growing by almost 15% per year on average, exceeding GDP of about 10% per year.

Table 4 presents ratios of key indicators. The ratio in the first column is the annualized growth in GGHE-D relative to GDP. In all cases, it is positive, but it is highest for China at 8.1% and lowest for India at 0.9%. The growth in GGHE-D relative to GGE is positive in all groups, meaning that as GGE grows, either the share going to GGHE-D grows faster or higher spending governments are allocating a higher share to health. This growth rate is highest for China.

The growth in GGHE-D relative to current health expenditure is relatively low but positive, except in the low-income group when India is excluded, where it is weakly negative. As mentioned previously, the growth of out-of-pocket spending relative to current health expenditure is negative in most groups, except for low-income groups once India is excluded, where it is close to zero.

Even among HICs, GGHE-D is growing faster than GDP and faster than GGE. Many HICs have achieved UHC or close to it, but demands for health, changes in real costs, technological change, and difficulties in cost containment can mean expenditures continue to rise faster than income growth. These challenges have been long-discussed, including by William Baumol in the early 1990s ^12^.

### Comparing financing trends from 2000-2015 with health spending targets

Targets have been set for certain expenditure indicators in international forums and in international reports. For example, in April 2001, the heads of state of African Union countries pledged to allocate at least 15% of government budgets to health. Less often quoted from the Abuja meeting, but also important, was the call on donor countries to meet the target of at least 0.7% of GNP to official development assistance to developing countries (although the proportion to health was left ambiguous) ^13^. Although there have been arguments about the somewhat arbitrary basis of “Abuja” target of 15%, it has been widely referenced since this meeting in discussions about resource mobilization. Considerable emphasis is given to the policy lever of reallocation of overall government expenditures towards health in domestic resource mobilization discussions. As an example of another target, the 2010 World Health report suggested that over the long term, the level of combined government spending and compulsory insurance (which we include in public spending in our analysis) needs to reach 5 to 6% of GDP to achieve UHC ^3^.

Figure 4 shows government expenditure on health (from domestic and external sources) as a share of government expenditure. While some individual countries reached the target, the LICs and LMICs income groups are very far from the Abuja target in 2015 (i.e., allocation of 15% of the government budget to health). As shown in Figure 4, between 2000 and 2015, this share increased from 4.8% to 5.2% for LICs and 8.6% to 10.2% for LMICs. The share for HICs (not shown in Figure 4) was over 20% in 2015. However, in many cases in HICs, *cost containment*, not resource mobilization, is high on the policy agenda, given what may appear to be unsustainable growth in government expenditures on health, both in absolute terms and as a share of government expenditure. Based on existing trends of health financing, while resource mobilization for health is an important priority for middle income countries, measures to improve efficiency (including cost containment) could also help to ensure that resources are allocated in an efficient manner across the sector. It should also be noted that the GHED provides data on total capital health expenditure and does not disaggregate public and private sources of capital expenditure. The GGHE/GGE somewhat underestimates what the number would be if public capital expenditure on health were included in the numerator. However, if all capital expenditure were added to the numerator, the finding still holds that LICs, LMICs, and UMICs are well below the Abuja target in 2015.

China, when it is disaggregated from the LMICs as a group, is rapidly increasing the share of health in its government expenditures. Health expenditures expanded from 6.2% to 10.1% from 2000 to 2015. This ratio has stagnated in LICs and UMICs. It has climbed in LMICs as a group, in part because of China’s impact on the estimates for this group.

Similarly, apart from HICs, no other income group has met the target suggested in the 2010 World Health Report of combined government spending and compulsory insurance reaching 56% of GDP. However, this ratio is growing in most income groups. GGHE as a share of GDP rose slightly in LICs from 1.1% to 1.3% between 2000 and 2015. India brings down the estimate for LICs. After removing India, this share increases to 1.5% for LICs but is still much lower than the recommended level ( Figure 5).

Our study found some outlier countries where the level of general government health expenditure as a share of both GDP and general government expenditures seemed very low or very high. In some cases, the change (increase or decrease) in GGHE/GDP and GGHE/GGE between 2000 and 2015 was very dramatic. These ‘outlier’ countries are listed in Appendix 4 ^6^. On the one hand, the performance of these countries may have varied greatly from the average values due to unique country experiences. On the other hand, data outliers can also point out possible data errors.

### Key drivers of growth for the increases in GGHE-D from 2000 to 2015

With the GHED database, we can identify how much of the growth in GGHE-D, in real terms, from 2000 to 2015 is due to (i) economic growth alone, (ii) expansion in public spending measured by GGE/GDP, and (iii) reallocation of GGE towards health measured by GGHE-D/GGE.

**Figure 6.**
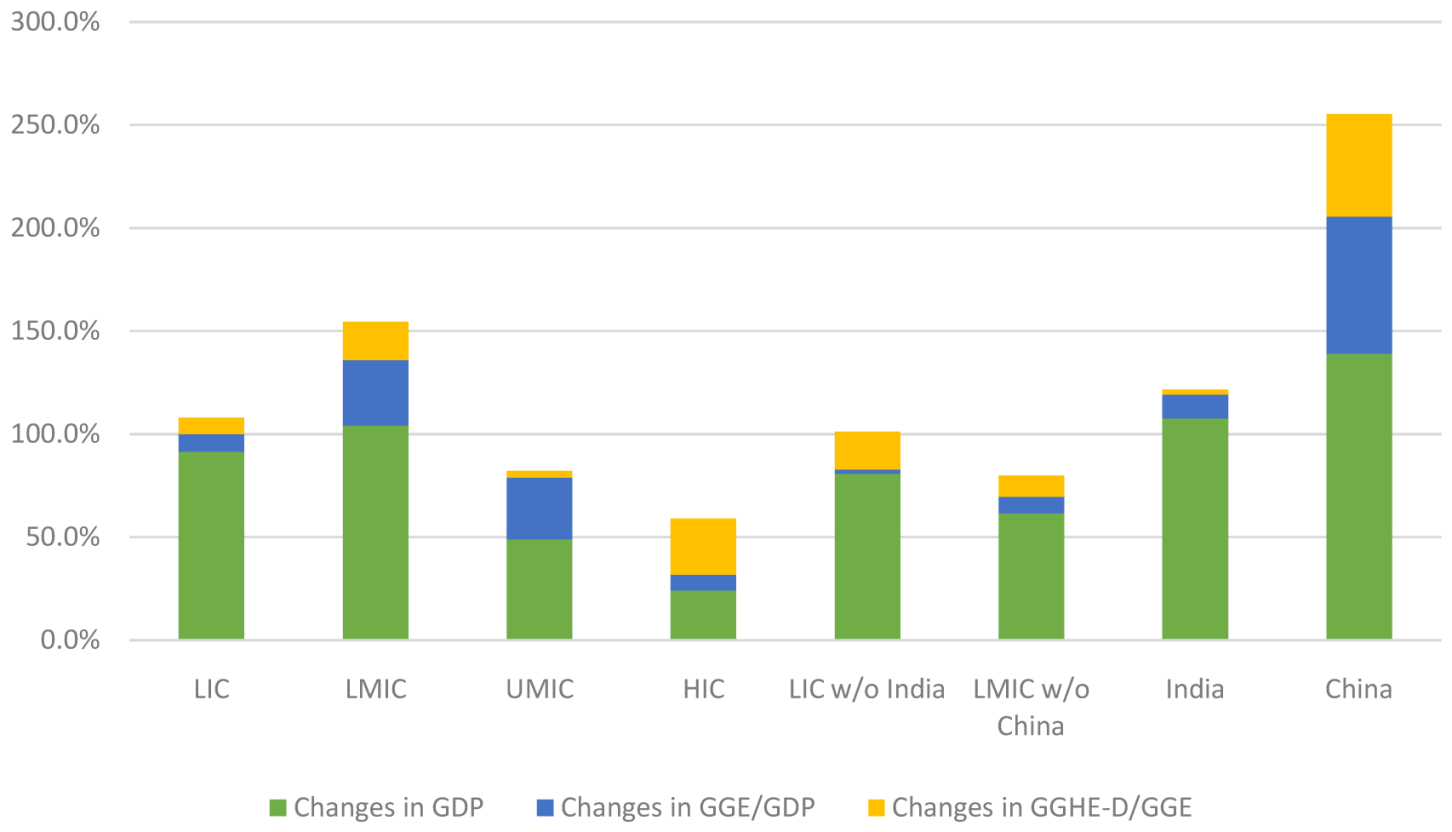
Decomposing increase in domestic general government expenditure on health (GGHE-D) from gross domestic product (GDP) growth, expansion in general government expenditure GGE/GDP, and reallocation of GGE for health.

Figure 6 shows the decomposition of change in GGHE-D between 2000 and 2015. Economic growth is the largest driver of increases in GGHE-D in all income groups, except HICs. An illustration of the decomposition of the growth in GGHE-D for LICs is shown in Table 5. In HICs, shifts in public spending towards health are also big drivers.

**Table 5.** Illustration of GGHE-D decomposition for low-income countries.

Indicators		2000	2015
**GDP (in 2010 constant US$, millions)**	**Y**	2,147,099.1	5,352,788.9
**GGE/GDP**	**E**	22.6%	24.5%
**GGHE-D/GGE**	**H**	4.4%	4.8%
**GGHE-D**	**G**	21,479.8	63,651.65
**Rate of growth in GDP**	**y**	150.8%	
**Rate of growth in GGE/GDP**	**e**	9.1%	
**Rate of growth in GGHE-D/GGE**	**h**	8.3%	
**Rate of growth in GGHE-D**	**g**	196.3%	
	**ln(1+y)**	0.91%	
	**ln(1+e)**	0.08%	
	**ln(1+h)**	0.08%	
	**ln(1+g)**	1.09%	
**Percent of change due to GDP growth**		84.0%	
**Percent of GGHED-D change due to change** **in GGE/GDP**		7.0%	
**Percent of GGHE-D change due to change** **in GGHE-D/GGE**		8.0%	

GDP, gross domestic product; GGE, general government expenditure; GGHE-D, domestic general government health expenditure.

Between 2000 and 2015, China experienced rapid economic growth with a 300% expansion of the GDP. Economic growth was accompanied by budget expansion, and rapid prioritization of health which is reflected in the GGHE-D decomposition for China shown in Figure 6. Once China is separated from the LMIC country group, it is seen that economic growth was the main driver of growth in GGHE-D in the LMIC without China group.

## Discussion

This study draws on data over the period 2000 to 2015 to examine how the composition of current health expenditure has changed, how health expenditures grew from different sources of financing, and what the main drivers of growth in GGHE-D were over this period. It presents results by broad income groups, with countries classified into groups by their income level at the start of the period, in contrast to the WHO ^3^ and World Bank ^4^ studies, which classified countries by their status at the end of the period for their analyses of the same period.

The study finds that LICs, taken as a group, were left out of the significant shifts that occurred in other income groups in the share of GGHE-D and OOP in CHE from 2000 to 2015. These shifts were largest for LMICs and UMICs. In LMICs, GGHE-D rose from 41 to 59% of CHE and OOP fell from 47 to 34%. In UMICs, GGHE-D rose from 32 to 57% of CHE and OOP fell from 14 to 12%. For HICs, GGHE-D grew from 55 to 59% as a share of CHE and OOP fell from 16 to 12%. However, in LICs, GGHE-D only rose slightly, from 28 to 29% of CHE and OOP expenditure fell only from 60 to 58% as a share of CHE. By the end of the period, the share of GGHE-D in CHE was twice as high in LMICs compared to LICs. LICs’ high share of OOP expenditure indicates little progress on financial risk protection over the period. Its low share of GGHE-D indicates little progress towards universal health coverage.

Growth rates of CHE p.c. and GGHE-D p.c. were fastest in LICs and LMICs over the period. By the end of the period, GGHE-D p.c. for LICs was US$20 p.c., up from US$9 in 2000. GGHE-D p.c. was $200 in 2015, up from $47 in 2000 (all absolute figures in 2010 US$).

Given the importance of GGHE-D in universal health coverage, we attempted to disaggregate the main drivers of GGHD-D growth over the period. We wanted to understand how much of the growth is due to reallocation of government spending towards health in the government budget, which is something that gets much attention in policy discussions. We wanted to understand how expansion in the size of the government contributed to increased resources for health. And we wanted to understand the impact of economic growth. We developed a methodology to quantify these three components to growth in GGHE-D based on these variables.

We find that economic growth was the largest single driver of increases in GGHE-D in almost all income groups, except HICs. Although overall growth in GGHE-D was the smallest for HICs, the change in GGHE-D due to economic growth was relatively lower and the largest driver was a greater share of GGHE-D out of government spending. In presenting our results, we also showed the findings for India and China separately. India was classified as an LIC at the start of the period, and China was classified as an LMIC. Both countries experienced rapid income growth but had divergent experiences in health expenditures.

The increase in government spending as a share of GDP and prioritization of health within the government budget were key in explaining the difference between China and India’s experience from 2000–2015. China’s increase in GGHE-D came not only from economic growth and growth in the share of GGE in relation to GDP, but also through prioritizing health within the government budget. India’s growth in GGHE-D, however, was primarily due to economic growth.

### Methodological approach

This study was possible because of the major changes to the WHO GHED in the December 2017 release. Our study reports on what the data for the years 2000 – 2015 show, while recognizing that the data, inevitably, contain errors and will change and improve over time. With the exception of China and India, our study reports on income group results, rather than individual country results, because the purpose was to understand broad trends. We used GGE/GDP as an indicator of the size of government spending in the economy. The results could vary significantly if data on tax revenues were used as expenditures can exceed revenues over some years. Our approach for disaggregating the sources of growth of GGHE-D into economic growth, changes in government spending relative to GDP, and reallocation within the government budget was also used by the World Bank study, although that study classifies income groups at the end of the period and presents country-weighted averages. Appendix 6 presents the country-weighted and expenditure-weighted averages for comparison.

Our study has focused on resource allocation and not on the important issue of improving efficiency. Funds for priority health activities can also be made available from improvements in allocative efficiency (spending money on the right things) and technical efficiency (spending money to achieve results at lowest cost), and fully using budgeted funds. Considerations of how to generate fiscal space for health at a country level ideally need both elements, revenue generation and efficiency measures.

### Comparison with other studies

WHO’s study New Perspectives on Global Spending for Universal Health Coverage draws on the same database as this study. However, because of differences in methodological approaches, some of the findings are different. The WHO study classified countries into income groups by their income status at the *end* of the 2000 to 2015 period, and we chose to classify countries into income groups by their income status at the *start* of the period. More importantly, the WHO study used country-weighted averages of indicators, while we calculated indicators for the income group as a whole, so larger countries have a larger weight in our results, and smaller countries have a smaller weight. As an example of how the results differ because of these two differences in methodology, the WHO study found that OOP expenditures as a share of CHE fell from 46% to 38% over the period for LICs, while we found only a slight drop, from 60 to 58% (and the numbers are much higher). Differences in results between the two studies are not surprising, given that the WHO study used country-weighted averages while we calculated results for the income group as a whole, and also because countries were classified into income groups at different points in time. India is included in our LIC group because it was classified that way in 2000, while India is not in the WHO LIC definition, because it was no longer an LIC at the end of the period. India has a large weight in our calculations because of its large size, but India would be given the same weight as any other country in the WHO results. In terms of broad conclusions, there is more similarity across the two studies. The WHO study, our study as well as the World Bank study Intertemporal Dynamics of Public Financing for Universal Health Coverage: Accounting for Fiscal Space Across Countries and others. The WHO study, our study, and others ^14, 15^ stress the importance of economic growth in driving GGHE-D growth relative to reallocation within the government budget. Looking forward, given the importance of economic growth in domestic resource mobilization, there is cause for concern in the poorer countries with slow or no growth projections and in conflict/fragile countries. Even in the poorer countries with rapid economic growth projections, it will take time to increase government budgets significantly given low starting points. External assistance for health needs to focus as much as possible on the poorest countries, and LICs and LMICs with unfavorable economic prospects.

## Conclusions

Our study found that economic growth was the largest driver of growth in GGHE-D in LICs, LMICs, and UMICs over the period 2000 to 2015. While growth in CHE p.c. and GGHE-D p.c. was fastest in LICs and LMICs, the composition of CHE changed the least in LICs. In LMICs and UMICs, the share of OOP in CHE fell and the share of GGHE-D in CHE increased significantly over the period. However, in LICs these indicators changed only slightly, indicating lack of progress towards UHC as measured by these indicators. With the exception of China and India, our study looked at income group aggregates, and individual country experience will differ. The appendices to this report ^4^ present country indicators. The outliers in country results could indicate distinct experiences or problems with the dataset (as this dataset is relatively new, and the quality of data do vary by country).

Discussions about a government’s ability to reallocate across government budgets need to be evidence based and pragmatic given the findings of the experience from 2000 to 2015. While there are exceptions, such as in China, most countries are moving very slowly towards prioritizing health more in government budgets. Our findings suggest that dialogue on domestic resource mobilization needs to be more balanced, with emphasis on overall economic growth and growth in the tax base as well as share of health in government budget, along with improving public financial management (not discussed in this paper). Arbitrary targets are unlikely to be helpful in these discussions.

This study reports primarily on results by country income group. A next step would be to delve into the differences within the groups to learn about important country differences in experiences and also to highlight possible errors in the data. The WHO GHED is an important public good and with further improvements in data accuracy over time, it will be an important tool for investigating important emerging trends of health expenditure around the world.

## Data availability

### Underlying data

Source data examined in this study, available in csv, xlsx and txt formats, have been collated on OSF. DOI: https://doi.org/10.17605/OSF.IO/WM8ZP ^6^.

### Extended data

Appendices associated with this study are available on OSF. DOI: https://doi.org/10.17605/OSF.IO/WM8ZP ^6^

Appendix 1. Change in composition of population across income groups between 2000 and 2015.

Appendix 2. Number of countries in World Bank analytical income classification categories based on GNI p.c. in 2000 and 2015.

Appendix 3. Trends in change in current health expenditure by country income group over 2000–2015.

Appendix 4. Outlier countries.

Appendix 5. Decomposition of the change in domestic general government health expenditure (GGHE-D) between 2000 and 2015

Appendix 6. Comparison of expenditure weighted and country-weighted average per capita values

Data are available under the terms of the Creative Commons Zero "No rights reserved" data waiver (CC0 1.0 Public domain dedication).
