# Supplementary material for: A quantitative analysis of sources of changes in government expenditures on health, 2000 to 2015: what can we learn from experience to date?
Source: Gates Open Res. 2021 Aug 26;3:5. Originally published 2019 Jan 11. [Version 2] doi: 10.12688/gatesopenres.12900.2 (PMC8408342; doi:10.12688/gatesopenres.12900.2)
Supplement: Peer review history

# Peer review: Reviewer response for version 2

**DOI:** 10.21956/gatesopenres.14312.r31148

**Published:** 2021-09-06

**Authors:** William Savedoff

**Affiliations:** 1 Inter-American Development Bank, Washington, DC, USA

I have read the revised paper and feel the authors have taken all the comments seriously. There is no single best answer to the weighting question and I respect the authors' choices now that the paper is quite explicit about the reasoning and consistent with interpretation.

 The paper is a very good contribution to the literature.

Is the work clearly and accurately presented and does it cite the current literature?

Partly

If applicable, is the statistical analysis and its interpretation appropriate?

Partly

Are all the source data underlying the results available to ensure full reproducibility?

Yes

Is the study design appropriate and is the work technically sound?

Partly

Are the conclusions drawn adequately supported by the results?

Partly

Are sufficient details of methods and analysis provided to allow replication by others?

Yes

Reviewer Expertise:

I have research experience in health economics, international comparative research, econometrics, and health systems research.

I confirm that I have read this submission and believe that I have an appropriate level of expertise to confirm that it is of an acceptable scientific standard.

# Peer review: Reviewer response for version 1

**DOI:** 10.21956/gatesopenres.13996.r26853

**Published:** 2019-04-23

**Authors:** Aparnaa Somanathan

**Affiliations:** 1 Health, Nutrition and Population Global Practice, World Bank, Washington, DC, USA

This paper contains very valuable analyses of trends and composition of government health spending, as well as key underlying factors. The context and motivation for this paper are clearly laid out: the push for UHC- both at global and country levels - and the transition from donor financing in many cases. In terms of public policy, the past decade have been quite significant for UHC with many low and middle income countries committing at a very high level to achieving UHC. It is helpful to be able to look at how public expenditures have fared during this period and whether they have lived up to the UHC rhetoric. The analysis and findings of this paper are therefore quite relevant to the future of this debate.. 

 I agree with the two other reviewers on the two substantive methodological issues they have raised:

1. The decision to use an expenditure-weighted series of indicators is problematic given that there is still quite a lot of heterogeneity within each income group even after excluding China, India and very small states. Each income groups contains countries with quite different public expenditure (general and health) trajectories and factors that drive those trajectories, making it difficult to draw meaningful conclusions about the group as a whole. I would agree with Bill Savedoff’s recommendation that the revised version include country-weighted versions from the same data to assess the influence of the weighting method on results. 
2. The decomposition method needs to reflect on the broader literature on this topic and why this method was chosen. 

High-income countries are singled out when arguing that difficulties in cost containment may be a reason why GGHE-D is growing faster than GDP. While GGHE-D increases in middle income countries may have occurred because of high level public policy commitments to achieving universal coverage, the evidence, by and large, shows that cost-containment is very much a problem. Country-specific analyses (e.g. WB analysis for Vietnam and Indonesia) suggest that the lack of cost containment is undermining progress towards UHC in spite of large increases in GGHE-D. It would be important to clarify this point, even if efficiency improvements are not the focus of this paper.

 Using increases in GGE as a proxy for strengthening the tax base and tax administration is not convincing. The authors do note the limitations of this method. I would recommend simply discussing changes in GGE and not extending the discussion to what it implies in terms of the tax base. For the LMIC group this is particularly problematic given the large number of resource-driven economies.

Is the work clearly and accurately presented and does it cite the current literature?

Yes

If applicable, is the statistical analysis and its interpretation appropriate?

Partly

Are all the source data underlying the results available to ensure full reproducibility?

Yes

Is the study design appropriate and is the work technically sound?

Partly

Are the conclusions drawn adequately supported by the results?

Partly

Are sufficient details of methods and analysis provided to allow replication by others?

Yes

Reviewer Expertise:

I have research experience in international comparative analysis of health financing systems

I confirm that I have read this submission and believe that I have an appropriate level of expertise to confirm that it is of an acceptable scientific standard, however I have significant reservations, as outlined above.

# Peer review: Reviewer response for version 1

**DOI:** 10.21956/gatesopenres.13996.r26856

**Published:** 2019-01-28

**Authors:** Ke Xu, Andrew Siroka

**Affiliations:** 1 World Health Organization, Geneva, Switzerland

The paper presented public spending on health from 2000 to 2015 and analyzed the causes by the changes in GDP, public spending and budget prioritization on health.

 The paper is very well structured and presented in a very reader-friendly way. The description of the methodology is very clear and easy to follow.

 We also appreciate the acknowledgement from the research team on WHO Global Health Expenditure Database. The highlighted outlines are also very helpful for the countries and WHO team to further investigate the improve the data quality.

 We would like to raise two methodological matters

1. The unit of the analysis
  On the unit of analysis, we agree with Bill Savedoff’s comment. Based on the research objectives the country as the analysis unit is more appropriate. Although the authors provided sensitivity test by excluding India and China, and excluded countries less than 600,000 population, still the difference in population size among countries are huge. For example, the population in Lao PDR is less than 7 million while in Bangladesh is about 160 million. We suggest the authors use unweighted estimation with country as the analysis unit.
2. The decomposition analysis
  We are not convinced that the method used for the decomposition analysis is desirable. The growth of GDP, GGE share in GDO and GGHED share in GGE are not linear addition. The GGHED increase is a production of GDP increase, GGE/GDP increase and GGHED/GGE increase. The equation would be as the following:
  gghed1/gghe0=(1+a)*(1+b)*(1+c)
  *a, b and c are the increase of the three parameters, respectively.*
  *ln(ffhed1)-ln(gghed0)=ln(1+a)+ln(1+b)+ln(1+c)*

Is the work clearly and accurately presented and does it cite the current literature?

Yes

If applicable, is the statistical analysis and its interpretation appropriate?

Partly

Are all the source data underlying the results available to ensure full reproducibility?

Yes

Is the study design appropriate and is the work technically sound?

Partly

Are the conclusions drawn adequately supported by the results?

Yes

Are sufficient details of methods and analysis provided to allow replication by others?

Yes

Reviewer Expertise:

Health economics, health expenditure, health financing policy

We confirm that we have read this submission and believe that we have an appropriate level of expertise to confirm that it is of an acceptable scientific standard.

# Peer review: Reviewer response for version 1

**DOI:** 10.21956/gatesopenres.13996.r26852

**Published:** 2019-01-14

**Authors:** William Savedoff

**Affiliations:** 1 Inter-American Development Bank, Washington, DC, USA

This paper describes changes in public health spending from 2000 to 2015 for country income groups, with separate analyses for India and China. It shows that public health spending grew significantly over this period in all income groups and mainly in association with rising national incomes. Lower Middle Income countries and Upper Middle Income countries had lower out-of-pocket spending and a rise in domestically-financed public health expenditure. Rising fiscal capacity (share of overall government spending in the economy) was only a significant factor in upper-middle-income countries; while policies reallocating public budgets toward health appear to have only been significant in high-income countries.

 The article offers a clear set of questions and sets out to answer them with appropriate data and methods.

 The article is clear and informative about the new database it employs, the country sample, and the methods of calculation. This should make it possible for readers to assess the results and interpret them appropriately.

 It further provides appropriate qualifications about the data and alerts readers to the fact that its scope is limited to questions of the level of resources used for health and does not encompass questions of how efficiently that money is spent.

 Nevertheless, I have several reservations about the paper related to its choice of weighting, the interpretation of the decomposition results, and lack of reference to the broader literature on health spending and economic growth.

*Choice of weighting*

 My main reservation about the paper is the decision to calculate indicators with country income-group aggregates, effectively choosing an expenditure-weighted series of indicators.

 The choice of weights is usually determined by the question that is being posed. If the question is about policy-making units of analysis, it would seem better to use country-weighted indicators. If the question is about the typical experience of a person in a given group of countries, then a population-weighted indicator would be better. I could think of reasons for choosing expenditure-weighted indicators as done by these authors, but none of those reasons seem relevant to the issues raised in the paper.

 Implicitly, the authors recognize the problems with their expenditure-weighting by taking India and China out of the income groups. However, this left me wondering, each time I read a finding, about the degree of heterogeneity that remains in each group. For example, after excluding India, the low-income country grouping results are probably more representative of Indonesia or Ukraine than Chad or Lesotho. A country-weighted indicator would avoid this problem by producing a mean effect across policy-relevant units of analysis, i.e. countries.

 If the authors choose to keep this weighting choice, then interpretations of the results should be consistent with this choice. For example, consider the following sentence:

 "While the results of the decomposition analysis can vary from an individual country to country basis, the reporting by income groups helps us identify whether *countries in general* have been creating more fiscal space for health by reallocating government budgets towards health, strengthening tax bases, or whether growing  government health expenditures are mostly spurred by economic growth alone. [italics added]"

 The phrase “countries in general” is somewhat misleading because it implies we have learned something about a typical country in this group. To be accurate, the sentence would have to replace "countries in general" with something like "countries in each income group when aggregated together" or "higher-spending countries in this income group."

 Similarly, the sentence on p. 11 offers the possibility that, “governments are allocating a higher share to health" when in fact the indicator shows only that *higher-spending governments* might be allocating a higher share to health.

 All of this may be of little importance if the country spending within each income group is relatively homogeneous. The authors helpfully presented graphs (Figures 4 through 6) in the same format as those in a WHO 2018 report which used country-weighted indicators. This allows readers to see that the trends look somewhat similar. However, that comparison is affected by more than just the weighting choice. In a revised version, the authors could give readers a sense of how much the expenditure-weighting approach affects the results by presenting more information about the divergence between their calculations and the WHO 2018 report or, better yet, by calculating the country-weighted version from their own data and assessing the influence of the weighting method on results.

*Decomposition method*

 The authors seek to measure the relative contribution of economic growth, fiscal capacity and budget allocations to the observed increase in government health expenditure. They do this with an identity that relates government health spending to GDP, government spending as a share of GDP, and government health spending as a share of total government spending. They then calculate three counterfactuals, each one a hypothetical government health spending level that changes one factor to its 2015 level while keeping the other factors at their 2000 levels.

 This method is one reasonable way to answer their question (i.e. how much do the changes in public health spending have to do with economic growth relative to public choices about taxation and spending allocations?). However, the paper presents the method as if it were an obvious choice and as if a larger literature trying to address this question does not exist. The same question has been addressed by simulations, statistical analyses, and other decompositions in the literature (see references below). Why was this particular approach chosen?

 The authors may also have mischaracterized the residual category in the decomposition analysis. The paper talks about this residual as if it were due to "multiplier effects" – which is not true (at least not in the macroeconomic sense) – and "interactions" – which is somewhat vague. At root, I suspect the residual is actually due to an index problem, not unlike the indeterminacy of inflation calculations using Paasche and Laspeyres indices. I believe that works by Das Gupta cited below (1991 ^1^ and 1993 ^2^) explain why such a decomposition for more than two factors is necessarily inconsistent. My understanding is that when variables change in small increments, the decomposition will successfully and intuitively account for a large share of the overall changes. However, when variables change in large amounts, the inconsistencies in the decomposition effectively swamp the utility of the calculation. If I'm right, then since some countries experienced massive transformations over this period and others much less, the ability to make judgments regarding the relative importance of the underlying factors is compromised. For example, the main thing that is apparent when comparing India and China is the large difference in the residual category which makes it difficult to say much about the relative amounts that can be associated solely with growth or budget changes. By aggregating countries into large income groups, the problem of the residual is attenuated but at the cost of masking heterogeneity within those groups.

*Health spending and national income*

 Despite investigating the widely-debated relationship between national income and health spending, the paper only mentions one theory on this topic, that of William Baumol. However, Baumol's work is only one of many on this topic and whether or not his argument applies to health spending (where productivity has, indeed, been growing by many measures) is contested. More relevant references that could inform the interpretation of results include Dormont et al 2010 ^3^; Fan and Savedoff 2014 ^4^; Gerdtham and Jönsson 2000 ^5^; Smith et al. 2009 ^6^; and Ke et al. 2011 ^7^; among others.

 If the authors choose to revise the paper, these are some smaller issues that they might want to consider addressing as well: 

- I found Figure 1 to be very confusing. Since these are accounting definitions, it should be possible to present them in a clearer hierarchical ordering. The position of boxes and the directions of arrows did not make sense to me.
- The authors might want to alert readers that the term “domestic general government health expenditure” encompasses more than direct spending by the government through a ministry of health or national health system. It also includes spending by social security institutions, publicly-financed sickness funds, and compulsory contributions to non-governmental institutions. Figure 1 lists the codes associated with these different categories (FS.1, FS.3, FS.4), but most readers will not know the coding systems. It would only take a sentence to explain and could forestall potential confusion.
- I believe the section comparing health financing indicators with targets leads policy discussions down a dangerous path and would recommend simply eliminating it. There is no technical basis for setting the Abuja target or the WHO “share of GDP spent on health” figure. The Abuja targets can lead to absurd policy conclusions (e.g. a country can look better on the health budget share by making draconian cuts in pensions and education). Furthermore, the failure to reach a certain share of GDP contains no particular information at all (see Savedoff 2007 ^8^ for an explanation of the origin of this WHO "target" and why the measure is not helpful).

Is the work clearly and accurately presented and does it cite the current literature?

Partly

If applicable, is the statistical analysis and its interpretation appropriate?

Partly

Are all the source data underlying the results available to ensure full reproducibility?

Yes

Is the study design appropriate and is the work technically sound?

Partly

Are the conclusions drawn adequately supported by the results?

Partly

Are sufficient details of methods and analysis provided to allow replication by others?

Yes

Reviewer Expertise:

I have research experience in health economics, international comparative research, econometrics, and health systems research.

I confirm that I have read this submission and believe that I have an appropriate level of expertise to confirm that it is of an acceptable scientific standard, however I have significant reservations, as outlined above.

## References

[1] : Decomposition of the difference between two rates and its consistency when more than two populations are involved. *Mathematical Population Studies*.1991;3(2) : 10.1080/08898489109525329105-12510.1080/08898489109525329

[2] : Standardization and decomposition of rates: A user's manual (No. 186). *US Department of Commerce, Economics and Statistics Administration, Bureau of the Census*.1993;

[3] : Health expenditure, longevity and growth. *Ageing, Health and Productivity: The Economics of Increased Life Expectancy: P Garibaldi; J Olivera Martins; J van Ours. Oxford University Press*.2010;

[4] : The health financing transition: a conceptual framework and empirical evidence.*Soc Sci Med*.2014;105: 10.1016/j.socscimed.2014.01.014112-2110.1016/j.socscimed.2014.01.01424524906

[5] : Chapter 1 International comparisons of health expenditure: Theory, data and econometric analysis.2000;1: 10.1016/S1574-0064(00)80160-211-5310.1016/S1574-0064(00)80160-2

[6] : Income, insurance, and technology: why does health spending outpace economic growth?. *Health Aff (Millwood)*.28(5) : 10.1377/hlthaff.28.5.12761276-8410.1377/hlthaff.28.5.127619738242

[7] : The determinants of health expenditure: a country-level panel data analysis. *Geneva: World Health Organization*.2011;

[8] : What should a country spend on health care?. *Health Aff (Millwood)*.26(4) : 10.1377/hlthaff.26.4.962962-7010.1377/hlthaff.26.4.96217630438
